# The homolog of Ciboulot in the termite (*Hodotermopsis sjostedti*): a multimeric β-thymosin involved in soldier-specific morphogenesis

**DOI:** 10.1186/1471-213X-10-63

**Published:** 2010-06-08

**Authors:** Shigeyuki Koshikawa, Richard Cornette, Tadao Matsumoto, Toru Miura

**Affiliations:** 1 Graduate School of Environmental Science, Hokkaido University, Sapporo 060-0810, Japan; 2 Laboratory of Molecular Biology, University of Wisconsin-Madison, Madison, WI 53706, USA; 3 National Institute of Agrobiological Sciences, 1-2 Ohwashi, Tsukuba, Ibaraki 305-8634, Japan; 4 Faculty of Liberal Arts, The Open University of Japan, Mihama-ku, Chiba 261-8586, Japan

## Abstract

**Background:**

Caste differentiation in social insects is a type of polyphenism that enables division of labor among members of a colony. This elaborate social integration has attracted broad interest, although little is known about its regulatory mechanisms, especially in Isoptera (termites). In this study, we analyzed soldier differentiation in the damp-wood termite *Hodotermopsis sjostedti*, focusing on a possible effector gene for caste development. The gene for an actin-binding protein, *HsjCib*, which shows a high level of expression in developing mandibles during soldier differentiation, is characterized in detail.

**Results:**

To examine the *HsjCib *gene, full-length cDNAs were obtained by rapid amplification of cDNA ends-polymerase chain reaction (RACE-PCR) and sequencing. Multiple isoforms were identified, and on the basis of the results of northern and Southern hybridization analyses, these isoforms were considered to be transcriptional variants from a single gene. On the basis of their sequence similarity to homologous genes of other organisms, functions in actin assembly were assumed to be different among isoforms. Expression analysis revealed high expression in the head during soldier differentiation, which was consistent with their allometric growth. Although isoform expression was observed in various tissues, different expression levels were observed among tissues, suggesting the possibility of tissue-specific morphogenetic regulation by *HsjCib *isoforms.

**Conclusion:**

This study revealed the characteristics and dynamics of the *HsjCib *gene during soldier differentiation as a potential representative of downstream effector genes in caste-specific morphogenesis. From the expression patterns observed, this gene is considered to be involved in cephalic morphogenesis and neural reorganization, resulting in the establishment of caste-specific morphology and behavior.

## Background

Social insects constitute complex societies with caste differentiation, and in some cases, they form huge colonies with vast numbers of individuals. The elaborate integration of insect societies has intrigued researchers for many years [1]. Termites (Isoptera, Insecta), which flourish in abundance and diversity in tropical and temperate zones and constitute one of the major groups of social insects, also form complex societies that include various castes [2,3]. The majority of social-insect research has focused primarily on hymenopterans (ants, bees, and wasps). Because the social mode and regulatory mechanisms of termites are different from those of hymenopterans in many respects [4,5], termites can provide an essential source of information for understanding the general and common features of sociality [6-8].

The morphologies of termite soldiers are highly specialized [9] and their differentiation is regulated through mutual interactions with other nest mates [10]. Soldiers are typical examples of the combination of exaggerated morphology [11] and polyphenism [12]. The regulatory mechanisms of polyphenism have been studied in various aspects and such studies have spurred the development of a new research field that spans the boundary between ecology and developmental biology [13]. Recently, the molecular biological approach for the study of social insects such as the ant [14-16], honey bee [17-22] and termite [23-38], has become common. Little is known, however, about the developmental basis of morphological modifications among polyphenic castes. Utilization of the molecular developmental approach with termites, whose caste development involves large morphological modification, has its own advantages and peculiarities [39].

**Figure 1:**
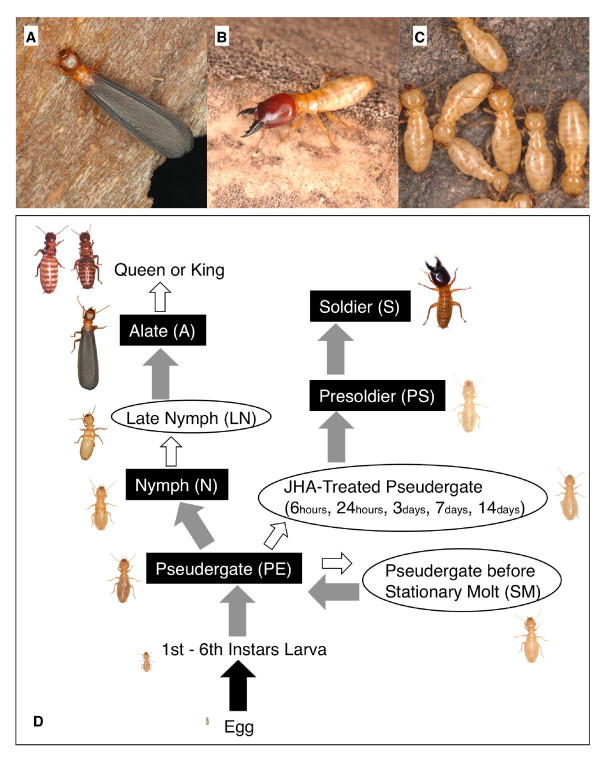
**Castes of the damp-wood termite *Hodotermopsis sjostedti***. **A: **Alate. **B: **Soldier. **C: **Pseudergates. **D: **Diagram of the caste-differentiation pathway, modified from Miura et al. [41]. The highlighted text denotes the castes used in the present study, and the non-highlighted, parenthesized letters are the short transitional stages used in the study. In the "JHA-Treated Pseudergate" category, we prepared several time stages after JHA application. Gray arrows indicate transitions with molting, white arrows indicate those without molting, and the black arrow indicates hatching.

The mode of postembryonic development and caste differentiation varies among termite lineages [5]. In the damp-wood termite *Hodotermopsis sjostedti*, the pseudergate (seventh or older instar apterous individual) has the potential to develop into one of several castes [6,40,41] (Fig. 1). Although a genetic factor is known to influence caste determination in the termite species *Reticulitermes speratus *[42], in termites in general it is recognized that environmental factors during larval or pseudergate periods determine the developmental fate of particular castes [10].

Gene expression is believed to be altered before the molt in particular castes, in preparation for specific morphologies. Juvenile hormone (JH) has been considered to physiologically control caste differentiation [43]. In many termite species, application of JH (including JH analogs [JHA]) results in differentiation into the soldier form. And thus, the application of JH (or JHA) is a useful mechanism by which to investigate caste differentiation [44,45].

Among termite lineages, a variety of soldier morphologies are known, which can be classified into several types. These types include the biting soldier, reaping soldier, snapping soldier, or nasute soldier [9]. The focal species in the present study, *H. sjostedti*, has a typical biting soldier morphology which is considered to be the most basic and primitive type among termites. During soldier differentiation, allometric growth of the whole head and mandibles has been observed [46]. Before the molt, a specific morphogenesis causes complex folds to be formed in the epidermal tissues inside the mandibles; this enables rapid expansion of the mandibles at the time of molt into presoldiers [47]. In a previous study, we screened the genes expressed in mandibles during soldier differentiation using the fluorescent differential display method [28]. *HsjCib*, a homolog of the *ciboulot *(*cib*) gene in the fruit fly *Drosophila melanogaster*, is one of the identified genes thought to be involved in morphogenesis. The *cib *gene encodes an actin-binding protein and is categorized as a multimeric β-thymosin [48,49]. It is known to be required for the central nervous modification during metamorphosis by genetic and developmental study [48,50] and to regulate actin polymerization by *in vitro *study [48]. In *H. sjostedti*, *HsjCib *in mandibles was the most abundantly expressed at the stage of 14 days after JHA application, which is when specific epidermal morphogenesis occurs.

Multimeric β-thymosins, including Cib, have homology with monomeric vertebrate Thymosin-β. Multimeric β-thymosin includes two-five WH2 domains (Wiskott-Aldrich syndrome protein homology domain 2--one of the actin-binding domains), while Thymosin-β includes only one WH2 domain [51-54]. Although their homology is relatively high, the functions of multimeric β-thymosins and those of Thymosin-β are quite different. Multimeric β-thymosins are thought to promote actin polymerization, whereas Thymosin-β is believed to sequester actin monomers and inhibit their polymerization. Hertzog et al. [55] discussed this distinction, and concluded that the amino acid residues at the N terminus of the WH2 domain are responsible for the different molecular functions. When the amino acid residues at the structurally important site in Thymosin-β were replaced with those of Cib, the molecular function was changed from sequestering to assembly-promoting [55,56]. Hertzog et al. [55] also proposed a model to predict WH2 domain function in many proteins by checking the amino acid residues at the structurally important site. The possible mechanisms by which structural differences translate to functional differences have been discussed previously by several authors [57-59].

Caste differentiation and polyphenism are often regulated through JH signaling, although the mechanisms that link JH signaling and morphological modification remain largely unknown. Characterization of the molecules involved in these mechanisms will contribute to the understanding of the regulation of polyphenism. In the present study, we focused on *HsjCib*, whose partial sequence was previously identified [28] as a gene responsible for morphogenesis during soldier differentiation in response to JH (JHA). We cloned the full-length cDNA of *HsjCib *and found multiple isoforms that may serve two different molecular functions. Here, we reveal the expression dynamics of *HsjCib *using quantitative real-time polymerase chain reaction (qPCR) and *in situ *hybridization and discuss the possible functional differences among isoforms.

## Results

### The full-length cDNA sequence and the number of WH2 domains

**Figure 2:**
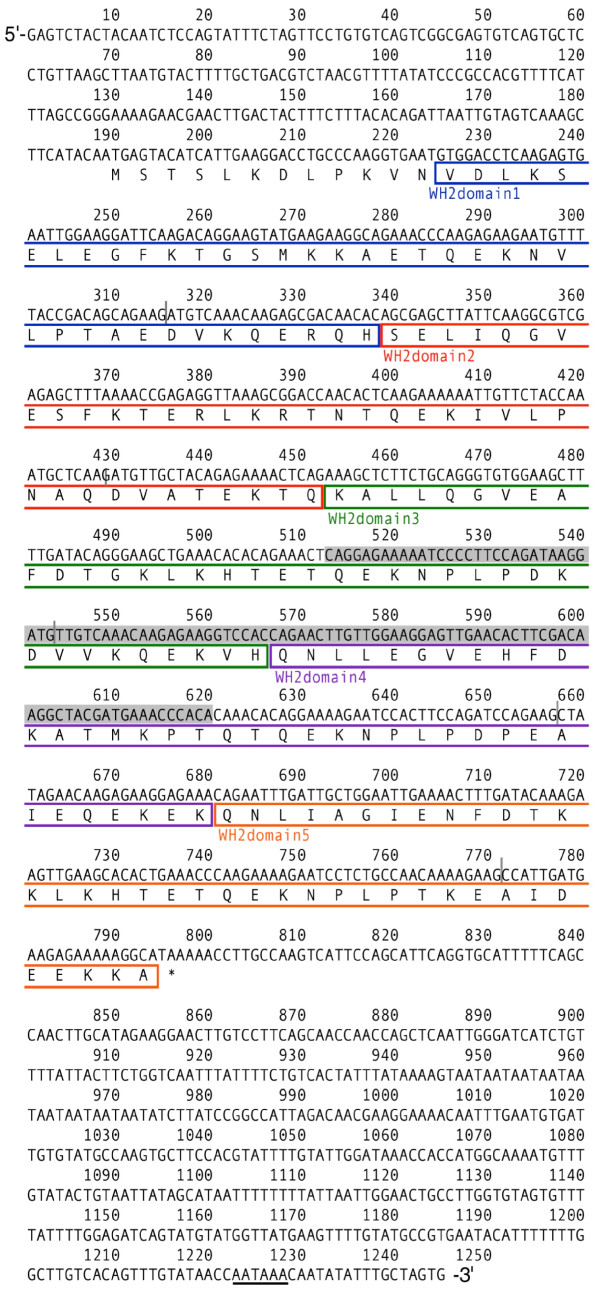
**cDNA and putative amino acid sequences for the longest isoform of *HsjCib***. The 1245-bp cDNA, obtained by 5' and 3'-RACE, contains a putative ORF that encodes a polypeptide of 202 amino acid residues. Boxed sequences indicate putative WH2 domains with a different color for each domain. The gray vertical bars indicate positions of introns. The gray shadow indicates a fragment from the original differential screening [28]. A potential polyadenylation signal is underlined.

**Figure 3:**
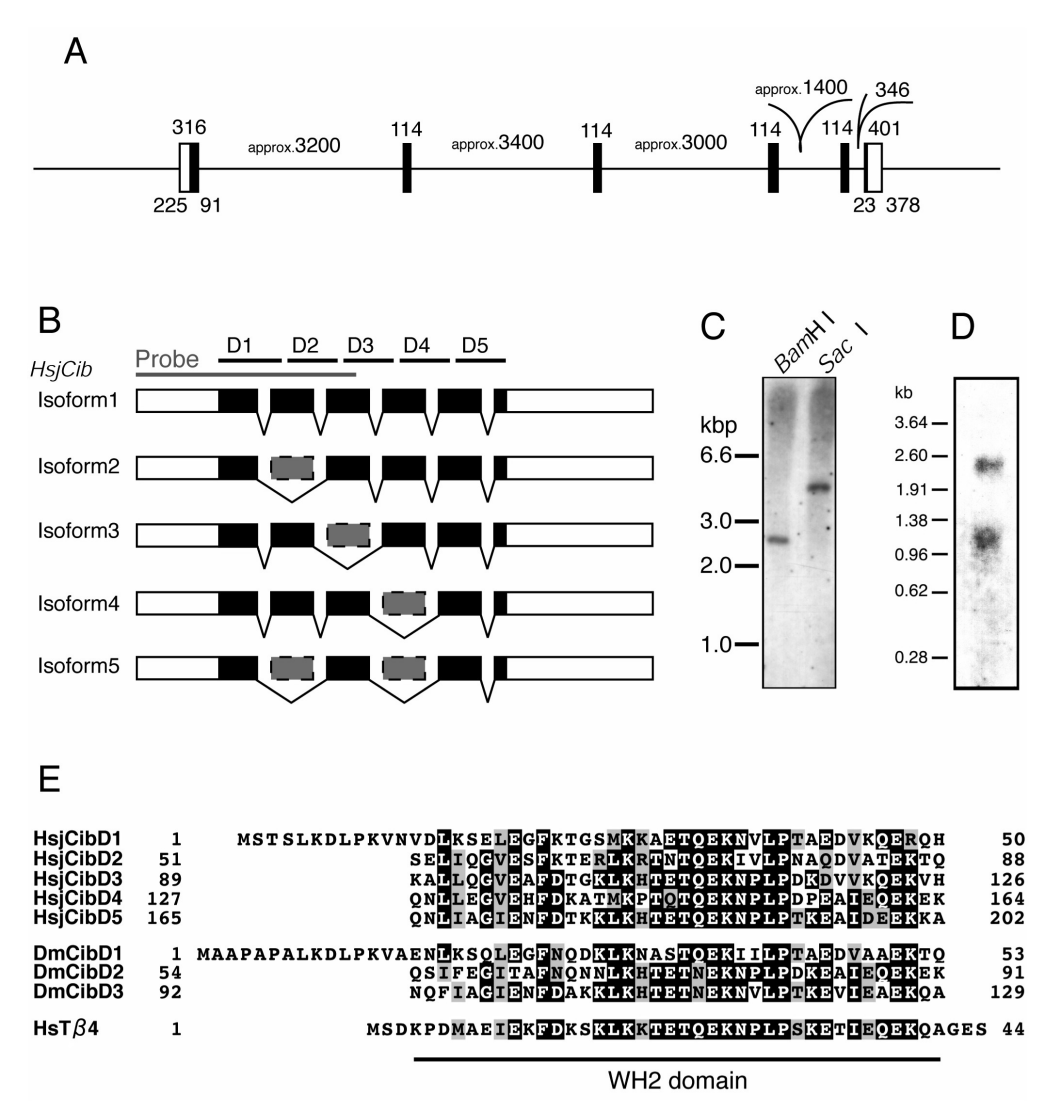
**Isoform structure of *HsjCib *gene**. **A: **The gene structure of *HsjCib *inferred from PCR amplification of genomic DNA. Coding regions are represented in black. Numbers indicate the lengths (bp) of regions. **B: **Structure of obtained isoforms. Smaller isoforms were thought to skip some exons. The gray bar indicates the fragment used as probes in Southern, northern, and *in situ *hybridization. D1 to D5 indicates putative WH2 domains. **C: **Southern hybridization indicates that *HsjCib *exists as a single-copy gene on the genome. **D: **Northern hybridization for *HsjCib*. Several isoforms seem to overlap between the 0.96- and 1.38-kbp markers. The longer fragment around 2.5 kb may be a paralog or an isoform that has not been cloned. **E: **Alignment of putative amino acid sequences of HsjCib with *Drosophila *Cib and human Thymosin-β4.

On the basis of the partial sequence of *HsjCib *previously identified by fluorescent differential display [28], we amplified both ends of the cDNA using the rapid amplification of cDNA ends (RACE) method and sequenced the full-length cDNAs of *HsjCib *(Fig 2). The predicted amino acid sequence of the longest HsjCib isoform was compared to other organisms with known sequences possessing WH2 domains and found that HsjCib contained five WH2 domains (Fig 2, 3A and 3E). The numbers of WH2 domains in known genes vary among organisms: *Drosophila *Cib, 3; *Caenorhabditis *Tetrathymosin, 4; the sea slug *Hermissenda *CSP24/29, 4/5; and a sea squirt *Ciona *Multimeric β-thymosin, 5 [48,49,59,60]. The number of WH2 domains in insects varies from three to five, the evolutionary origins and history of duplication/deletion being unclear (see Additional file 1 and 2). The functional significance of the number of WH2 domains is still open to debate [55,57,59].

### The structure of isoforms

Five *HsjCib *isoforms were identified from cDNA cloning and sequencing. Four isoforms were truncated compared to the largest isoform (Fig. 3A and 3B). Of the five isoforms, three had the same length: one 1245-bp isoform, three 1131-bp isoforms, and one 1017-bp isoform (accession nos. AB534909, AB534910, AB534911, AB534912, AB534913). Exons 2, 3, 4, and 5 each had a length of 114 bp, which corresponded to 38 amino acid residues and was the same length as a WH2 domain. On the basis of the results of Southern hybridization, we considered the identified isoforms to be splice variants from a single gene (Fig. 3C). The skipping of exons during splicing predicted isoforms with five, four or three WH2 domains. The editing sites of the exons were located in the middle of the WH2 domains, forming a complete WH2 domain when two incomplete portions of WH2 domains were spliced together. By northern hybridization, the splice variants were observed to be poorly separated and a broad band was detected (Fig. 3D). Although another band was observed at around 2.5 kb, we could not conclude whether the band represented an uncloned large isoform, a paralog, or DNA carry-over. The approximate arrangement of exons was estimated using the PCR fragments from the genomic DNA (Fig. 3A). The borders between exons and introns were determined by sequencing both ends of the PCR fragments.

### Western blot analysis

**Figure 4:**
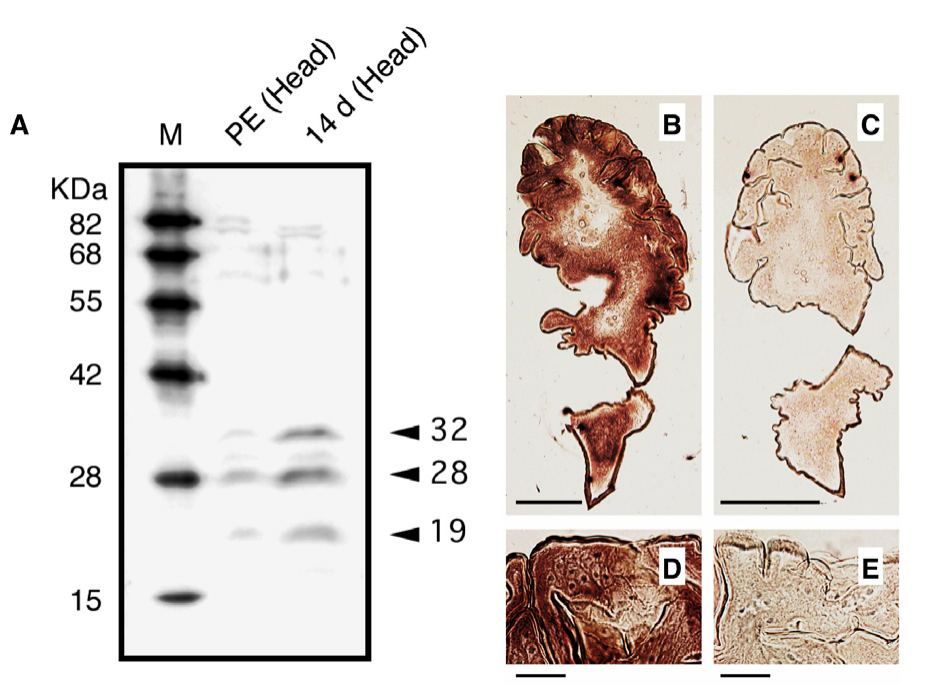
**Western blot and *in situ *hybridization to detect *HsjCib *expression**. **A: **Western blot analysis detected by anti-*Drosophila *Cib antibody against head homogenates of the termite. The antibody detected strong bands in the 14 d (14 days after JHA application) lane and weak bands in the PE (normal pseudergate) lane, which were presumed to correspond to isoforms of HsjCib. **B-E: ***In situ *hybridization of the *HsjCib *mRNA in newly formed mandible (14 d stage). Transverse paraffin sections (6 μm) were subjected to *in situ *hybridization with antisense (B and D) and sense (C and E) DIG-labeled RNA probes. Bars indicate 200 μm (B and C) and 20 μm (D and E), respectively. The expression of *HsjCib *was observed in the mandibular epidermis.

Because the expression of mRNA does not provide strict evidence of actual protein expression, protein expression was confirmed by western blot analysis (Fig. 4A). The results were consistent with the expectations for the three isoform sizes from cDNA cloning. However, the molecular weights estimated from relative mobility to markers (32, 28, and 19 kDa) were larger than those estimated from the predicted amino acid sequences (23, 19, and 14 kDa, as computed with the pl/Mw tool in ExPASy, http://ca.expasy.org/tools/). We considered two possibilities for the inconsistency between the electrophoretic mobility and predicted molecular weights. One possibility was that posttranslational modifications caused the difference in mobility. The homolog in *Hermissenda*, CSP24, is known to be phosphorylated [61], and several possible phosphorylation sites were found in the putative amino acid sequences of HsjCib (Net PhosK 1.0 server, http://www.cbs.dtu.dk/services/NetPhosK). No possible N-glycosylation site [N-X-S(T)] was found in the amino acid sequences (Fig. 2).

Although unlikely considering the size relationships, another possibility was that the three isoform sizes did not correspond to the three sizes of bands on the western blot. In that case, the 32-kDa band corresponded to an uncloned isoform and perhaps to the 2.5-kb band observed in northern hybridization. In this possibility, the smallest isoform (isoform 5) derived by cDNA cloning should have been scarcely expressed. However, cDNA of isoform 5 was frequently obtained during the cloning process. Because the isoform sequences resembled each other, it was unlikely that cross-reactivity of the antibody to the different isoforms was very different.

### *In situ *hybridization

**Figure 5:**
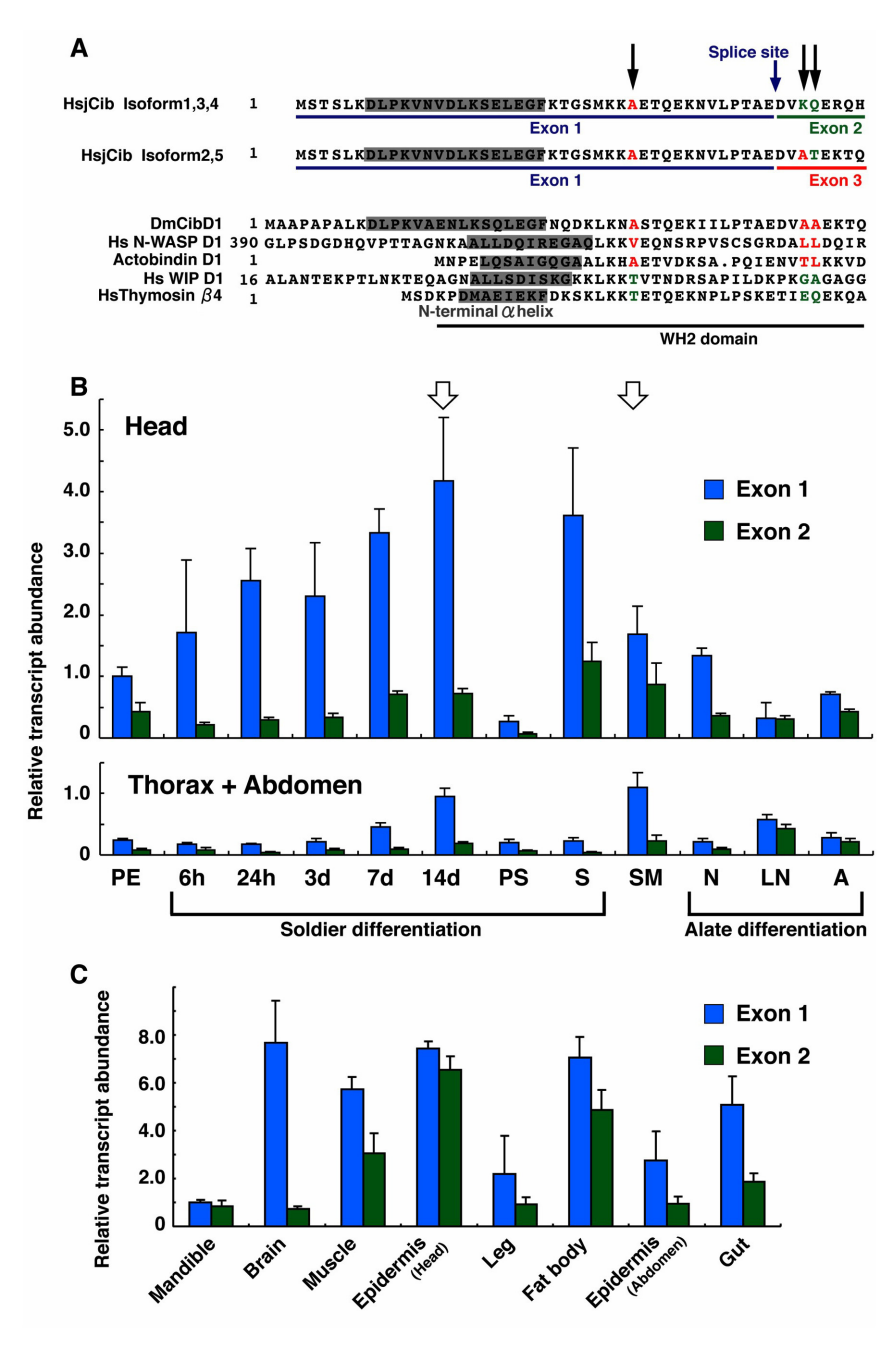
**Expression analyses of *HsjCib *in various stages and tissues**. **A: **Alignment of WH2 domains in various proteins. Black arrows indicate structurally important amino acid residues as suggested by Hertzog et al. [55]. According to the Hertzog's model, *HsjCib *isoforms 2 and 5 are supposed to act as assembly-promoting proteins, whereas isoforms 1, 3, and 4 resemble G-actin sequestering proteins. **B: **Temporal expression pattern of *HsjCib *during caste differentiation. Exon 1 was contained in all isoforms; consequently, the expression levels of exon 1 indicate the sum of the expressions of all isoforms. The expression levels of exon 2 were thought to indicate G-actin sequestering isoforms. PE: Pseudergate; 6 h-14 d: pseudergate of 6 h to 14 days after JHA application; PS: Presoldier; S: Soldier; SM: Pseudergate before stationary molt; N: Nymph; LN: Late nymph before imaginal molt; A: Alate. White arrows indicate two comparable stages in which the times until the next molt are approximately the same. **C: **Measurement of expression levels of exon 1 and exon 2 in the indicated tissues at 14 d after JHA application.

Because immunohistochemistry did not produce a distinct signal (the antibody used was known to be ineffective for immunohistochemistry, T. Préat, personal communication), we investigated *HsjCib *expression in the mandibular tissue by *in situ *hybridization using 473 bases of the RNA fragment transcribed as a probe *in vitro *(Fig. 3B). On cross sections of developing mandibles, we identified the *HsjCib *signal in epidermal tissue (Fig. 4). Although *HsjCib *was originally observed in the screening for mandibular expression, the expression was also observed in many tissues, as shown in the next section and in Fig. 5.

### Functional differences among HsjCib isoforms

HsjCib isoforms 1, 3, and 4 resembled the "sequestering form," since the functionally important residues in the N-terminal side WH2 domain [55] were hydrophilic K (lysine) and Q (glutamine) (hydropathy index -3.9 and -3.5 according to the calculation method by Kyte and Doolittle [62]). HsjCib isoforms 2 and 5 resembled the "assembly-promoting form" because the functional residues were A (alanine) and T (threonine) (hydropathy index 1.8 and -0.7), which were somewhat hydrophobic (Fig. 5A). These resemblances were not perfect, and we could not assert their precise functions. The features of the functionally important sites were different among *HsjCib *isoforms, meaning that their properties should have differed from each other. In the sections below, for simplicity, isoforms 1, 3, and 4 will be referred to as "sequestering types," and isoforms 2 and 5 will be designated "assembly-promoting types." This naming convention should aid in understanding the complicated expression analyses.

### Quantification of expressions by qPCR

To confirm the gene expression pattern, we performed qPCR for "head" tissues and "thorax + abdomen" tissues over the time course (temporal pattern) and for several tissues at the 14 d (14 days after JHA application) stage (spatial pattern). To detect the isoform joining of exons, we initially tried to identify every isoform separately by making primers on the editing points between exons and introns (see also Fig. 3B). This procedure, however, failed to discriminate each isoform separately in the preliminary experiments. Consequently, we discontinued quantifying all the isoforms separately, and instead designed primers to quantify exons 1 and 2 separately. Exon 1 was contained in all the isoforms, and its expression amounts were equal to those of all *HsjCib *isoforms (sequestering + assembly promoting). Exon 2 was contained in isoforms 1, 3, and 4. Since the existence of exon 2 makes a sequestering isoform, its expression amount apparently represents the amount of sequestering isoforms. With this experiment, note that castes (PE, PS, S, N and A) were not necessarily in a same intermolt stage, but use of pooled samples could average their expression levels.

Quantification of exon 1 in termite heads demonstrated that the expression level gradually increased during soldier differentiation; the highest expression was observed at the 14 d stage, just prior to the molt to presoldier (Fig. 5B). The expression level was subsequently reduced in the presoldier, but was again increased in the soldier. Exon 1 expression was also increased in the SM stage (pseudergate before the stationary molt), although the level was lower than that observed at 14 d. A comparison indicated that the days to molt were approximately the same between the SM and 14 d stages [28]. Expression level was not high before the imaginal molt.

Exon 1 expression levels can be correlated to allometrical changes associated with prospective growth [46,47]. It is notable that expression level of exon 1 was highest in the head at 14 d when morphogenesis was taking place. Thorax + abdomen exon 1-expression was generally lower than that of the head, although at 14 d and in the SM stage it was higher than expected. During soldier differentiation, expression of exon 1 was much higher in the head samples compared to thorax + abdomen. Expression levels were similar in both tissues while in stationary molt (Fig. 5B white arrows). The expression of exon 2 was much less than that of exon 1 during soldier differentiation, which meant that *HsjCib *isoforms without exon 2 (assembly-promoting type) were primarily expressed.

Future wing tissue rapidly grows inside the wing bud in the late nymph stage (LN) [41], but considering the active morphogenesis taking place, expression levels of exon 1 was not observed to be high. In LN, exon expression was not very different, which meant that *HsjCib *isoforms with exon 2 (sequestering type) were mainly expressed.

Quantification in various tissues at 14 d revealed *HsjCib *expression in every tissue that was examined (Fig. 5C). The exon transcript amounts indicated that the proportions of assembly-promoting type splice variant (amounts of exon 1 minus amounts of exon 2) and sequestering type splice variant (amounts of exon 2) were different among tissues. The largest proportion of assembly-promoting type splice variant was observed in the brain. This corresponded well to *Drosophila*, in which Cib is required in the brain during metamorphosis and for which only the assembly-promoting type is known. In *Hodotermopsis*, the behavioral pattern is greatly altered during soldier differentiation, where HsjCib is possibly involved in neuronal modification with axon growth. In mandible and head epidermis, the proportions of sequestering type variants were higher. Isoforms with prospective functions were expressed differentially among tissues, suggesting that HsjCib regulates cellular morphogenesis in diverse ways among various tissues.

## Discussion

This study demonstrates that the expression profiles of *HsjCib*, acts as a downstream gene in a regulatory pathway which correlates with tissue morphogenesis occurring in termite soldier differentiation. The genes underlying caste differentiation of social insects have been extensively studied, but the present study is the first report on the detailed expression properties of a possible effector gene for caste-specific morphogenesis.

### Putative function in soldier differentiation

Previously, we demonstrated the expression of *HsjCib *in mandibles during soldier development was approximately 20-fold higher than that in an intermolt (pseudergate) stage [28]. Recent experiments show an almost ubiquitous expression of the gene throughout the body. *HsjCib *expression levels in the thorax + abdomen during stationary molt was unexpectedly equivalent to that in soldier differentiation. Although it is not a specific gene to soldier differentiation, *HsjCib *is required for every molt.

Conspicuous morphogenesis in the epidermis and fat bodies before the presoldier molt of *Hodotermopsis *correlates closely to elevated *HsjCib *expression in the head as well as the thorax + abdomen at the 14 d stage [47,63]. In *Drosophila*, Cib is involved in neuronal modification during metamorphosis [48]. During developmental modification in the brain of *Hodotermopsis*, neuronal changes required for soldier behavior occur [64], implicating high *HsjCib *expression levels in the axonal growth, retraction or synaptic development during this stage.

Soldier differentiation is a peculiar developmental pathway which was acquired only once in the ancestral lineage of termites [5,39]. The present study showed that *HsjCib *has ubiquitous expression, but when considering its responsiveness to JHA treatment and linkage to presoldier-dependent morphogenesis, the JH-dependent elevation of Cib expression seems to have evolved in the lineage from an ancestral termite to *Hodotermopsis*. This supports the idea that a preexisting gene recruited for exploitation in soldier differentiation acquired a new regulatory mechanism and novel way of morphogenesis.

### Relationship between functions and sequence difference among isoforms

The expression level of the assembly-promoting type exon tended to be higher than the sequestering type. These proportional differences imply the control mechanism of these two types. Further molecular functional analysis of HsjCib would require protein purification or recombinant protein synthesis as well as subsequent *in vitro *analysis with actin molecules.

RNA interference (RNAi) is also a way to test the function of *HsjCib*. However, in our preliminary RNAi experiment did not provide clear results of phenotypic impacts (see Additional file 1).

### The role of HsjCib among cytoskeleton-regulating molecules

Actin is one of the fundamental cytoskeletal components; its assembly and disassembly are the most important processes of morphogenesis at the cellular level [65]. This mechanism was thought to be regulated by various actin-binding proteins [51]. In the present study, we analyzed one gene, *HsjCib*, as a downstream gene in caste differentiation in the termite. Multiple actin-binding proteins have been shown to regulate cellular morphogenesis in animals [51,66,67]. In the termite, other proteins, such as Profilin, ADF/Cofilin and CAP [51,67] in addition to HsjCib could be cooperatively involved in cellular morphogenesis.

### Morphogenetic regulation during soldier differentiation

Cytoskeletal regulators are influenced at multiple levels by various molecules. In our previous work, regulatory molecules such as G-protein-related factor RhoGEF and splicing factor U2AF were revealed to be upregulated in developing mandibles during soldier differentiation [28]. Considering that soldier differentiation in the termite is triggered by a high JH titer, these genes would be regulated downstream of the JH-signaling cascade. The intrinsic JH titer and histological aspects of hormone-producing glands have been studied extensively in the focal species [68]. Because the expression of *HsjCib *was already altered at 6 h after JHA application, it is conceivable that the expression was directly controlled by JH or through a small number of regulatory factors. Further analysis will shed light on the mutual relationships among these factors and the overall picture of soldier differentiation regulation.

In the black marching termite *Hospitalitermes*, which is phylogenetically distant from *Hodotermopsis*, soldier mandibles do not develop; instead, a horn-like structure called a "nasus" is formed during soldier differentiation [69]. Folding structures of the epidermis and their subsequent unfolding and growth after the molt into presoldier are common mechanisms in biting soldiers (e.g., *Hodotermopsis*) and nasute soldiers (e.g., *Hospitalitermes*) [47,69], suggesting that functions of various genes (including *cib *homolog) would be conserved. Extensive search with expressed sequence tag (EST) analysis, which has already been performed in *Reticulitermes *[31,38], will help to obtain a more comprehensive picture of this unique morphogenesis.

There is limited knowledge regarding the regulatory mechanism of the exaggerated morphology and phenotypic plasticity of insects [12,70-73]. The generalized question would be if there is a common molecular bases among independent lineages of species with exaggerated morphologies. The regulatory mechanism for phenotype and their connection to the subsequent processes of morphogenesis are important for understanding phenotypic evolution.

## Conclusion

The present study documents the characteristics of the termite homolog of an actin-binding protein gene, *HsjCib*, which is one of the downstream genes for soldier differentiation. HsjCib includes up to five WH2 actin-binding domains, and functional differences in actin polymerization among isoforms were predicted on the basis of their similarity to homologous genes of other organisms. During caste differentiation, multiple isoforms were expressed with different quantitative ratios in various tissues. We propose the high probability that the functions of these isoforms during soldier differentiation involve cephalic morphogenesis and neural rearrangement, enabling soldier-specific exaggerated morphology and defensive behavior.

## Methods

### Insects and RNA extraction

Colonies of *H. sjostedti *Holmgren (Isoptera, Termopsidae) were sampled from rotten wood in evergreen forests on Yakushima Island, Kagoshima Prefecture, Japan, from May 2003 to 2007. They were kept in the laboratory as stock colonies at approximately 25˚C under constant darkness. Total RNA was extracted from various castes and stages (Fig. 1D) determined by their morphology [28,40,41]. Soldier differentiation was induced by the ingestion method of a juvenile hormone analog (JHA, pyriproxyfen, Sumitomo chemicals) [45], and tissues were analyzed by RACE, northern hybridization, western blot and *in situ *hybridization. The topical application method of JHA [23] was used for tissue examination via qPCR. Individuals from one or two colonies were used for each experiment utilizing seven colonies in total.

### Cloning of full-length cDNA by RACE

Total RNA was extracted from the mandibles of 14 d pseudergates (14 days after JHA application) using an RNAgents Total RNA Isolation System (Promega). Total RNA was reverse-transcribed with oligo(dT) primer, and RACE-PCR was performed using a SMART-RACE Kit (BD Bioscience). The PCR product was subcloned into pGEM vector with a pGEM-TA Cloning Kit (Promega). Nucleotide sequence was determined with a BigDye Terminator v3.0 Kit and an ABI 310 Genetic Analyzer (Applied Biosystems).

### Southern hybridization

Genomic DNA was extracted from whole insects (without guts) using a Wizard Genomic DNA Extraction System (Promega). Extracted DNA was digested with restriction enzymes (*Bam*HI or *Sac*I) for 48 h, separated in an agarose gel, and transferred to a positive-charge membrane, Hybond N+ (Amersham). The genomic *HsjCib *allele was detected by an Alkphos Direct Labelling and Detection System (Amersham). The 473-bp DNA fragment was used as a probe, as indicated in Fig. 3B.

### Northern hybridization

Total RNA was extracted from 160 individuals of 14d, using an RNAgents Total RNA Isolation System (Promega). Poly(A) RNA was isolated from total RNA using a PolyATract mRNA Isolation System (Promega). Poly(A) RNA (23 μg) was separated in an agarose gel, and transferred to a positive-charge membrane, Hybond N+ (Amersham). *HsjCib *mRNAs were detected by an Alkphos Direct Labeling and Detection System (Amersham). The 473-bp DNA fragment was used as a probe, similar to Southern hybridization.

### Western blot analysis

The heads of each of two normal pseudergates and 14 d pseudergates were homogenized in lysis buffer, and the protein quantities were calibrated by optical density (OD) at 280 nm using a protein quantification mode of a NanoDrop ND-1000 spectrophotometer. Proteins were separated in a sodium dodecyl sulfate (SDS)-polyacrilamide gel and electroblotted onto a polyvinyl difluoride membrane (Cosmo Bio). The proteins bound to the membrane were probed with anti-*Drosophila *Cib rabbit antiserum at 1:2000 dilution [48] followed by an anti-mouse IgG conjugated with horseradish peroxidase at 1:2000 dilution. Bands were visualized by fluorography with an ECL Western Blotting Detection System (Amersham).

### *In situ *hybridization

The mandibles were dissected from 14 d pseudergates and embedded in paraffin, after which cross sections were made, as described by Koshikawa et al. [47], except that 4% paraformaldehyde was used instead of FAA for fixation and all steps were performed under RNase-free conditions. The target gene in the sections was detected using an ISHR Starting Kit (Nippon Gene). Briefly, the DIG-labeled RNA probe was prepared by *in vitro *transcription from a DNA fragment (473-bp DNA, as indicated in Fig. 3B, plus a T7 or SP6 promoter sequence) and hybridized to rehydrated sections. The signal was detected by an alkaline phosphatase-labeled anti-DIG antibody with NBT/BCIP solution as substrate.

### Quantification of expression levels among developmental stages

Total RNA was extracted from 14-20 heads and thoraxes + abdomens of various castes and transitional stages (Fig. 1D) with an RNAgents Total RNA Isolation Systems (Promega). Individuals used were essentially from a same colony to unify the genetic background. Reverse transcription was performed with random hexamers. The expression levels at different stages were quantified by qPCR with a Power SYBR Green PCR Master Mix and an ABI Prism 7000 Sequence Detection System (Applied Biosystems). As an endogenous control of constitutive expression, we used 18S rRNA gene sequences (AF220567). When β-actin was used as an endogenous control, results were similar to 18S rRNA (data not shown), but due to high expression in the soldier caste and inconsistent expression, 18S rRNA was the preferred control [74]. Primer sequences for qPCR were as follows: HsjCib-Exon1F, AGGACCTGCCCAAGGTGAA; HsjCib-Exon1R, CTTCCTGTCTTGAATCCTTCCAA; HsjCib-Exon2F, GACAACACAGCGAGCTTATTCAA; HsjCib-Exon2R, GAGTGTTGGTCCGCTTTAACCT; Hsj18S-F, CTTGCAATTGTTCCCCATGA; and Hsj18S-R, ACGTAATCAACGCGAGCTTATG. The baseline and threshold for the Ct (cycle threshold) were set automatically. Each category was tested in triplicate, and standard errors were calculated by the relative standard curve method as described in User Bulletin 2 for the ABI Prism 7700 Sequence Detection System (Applied Biosystems). Statistic significances were analyzed by one-way ANOVA and Tukey's HSD test (see Additional file 1 and 3).

### Quantification of expression level among tissues

Each of seven normal pseudergates and 14 d pseudergates was dissected for various specific tissues (mandibles, brain, mandible closer/opener muscle, head epidermis, leg, fat body, body epidermis, and gut), and total RNA was extracted from them with an SV Total RNA Extraction System (Promega). The quantification methods used were the same as those described above.

## Competing interests

The authors declare that they have no competing interests.

## Authors' contributions

SK carried out most of the experiments in this study. RC participated in experimental design and carried out some experiments. TMa participated in experimental design and coordination. SK and TMi were responsible for overall experiment design, analysis of data, and writing of the manuscript. All authors have read and approved the final manuscript.

## Supplementary Material

Additional file 1 **Supplemental results**. RNAi experiments, phylogenetic analysis of *cib *homologs and statistic analysis of qPCR. Click here for file

Additional file 2 **Supplemental Fig. 1**. Phylogenetic analysis of *HsjCib *and *ciboulot *homologs from other animals. Click here for file

Additional file 3 **Supplemental Table 1**. Summary of Tukey's HSD test for qPCR results. Click here for file
