# Supplementary material for: The homolog of Ciboulot in the termite (Hodotermopsis sjostedti): a multimeric β-thymosin involved in soldier-specific morphogenesis
Source: BMC Dev Biol. 2010 Jun 8;10:63. doi: 10.1186/1471-213X-10-63 (PMC2896938; doi:10.1186/1471-213X-10-63)
Supplement: Additional file 1 — Supplemental results. RNAi experiments, phylogenetic analysis of cib homologs and statistic analysis of qPCR. [file 1471-213X-10-63-S1.PDF]

Koshikawa et al. BMC Developmental Biology

Additional information (Additional file 1)

Phylogenetic relationships among Ciboulot/Multimeric β-thymosin homologs

HsjCib has five WH2 domains, which is unique in insects. To investigate its phylogenetic position among homologs, we aligned protein sequences and drew neighbor-joining (NJ) phylogenetic trees using Clustal W program at DDBJ (http://clustalw.ddbj.nig.ac.jp/top-e.html) with default settings of the program. Among the insect clade, *Hodotermopsis* HsjCib was positioned in a reasonable phylogenetic position (even though the relationships of holometabola were not entirely accurate comparing with recent major hypothesis), which means this protein appears to be an ortholog of Ciboulot/Multimeric β-thymosin, and to have arisen from an ancestral protein of all insect species (Supplemental Fig. 1A). An additional WH2 domain in HsjCib seems to be made by a duplication event of middle WH2 domains, because the most N-terminal domains and the most C-terminal domains were clustered together, indicating those domains were not duplicated during HsjCib evolution (Supplemental Fig. 1B). The genes used were as follows, similar to thymosin beta [*Tribolium castaneum*] XP_976221.1, similar to thymosin beta [*Acyrthosiphon pisum*] XP_001951088.1, thymosin [*Bombyx mori*] NP_001040486.1, thymosin beta [*Scylla paramamosain*] ACY66642.1, similar to thymosin beta [*Nasonia vitripennis*] XP_001603735.1, ciboulot [*Drosophila melanogaster*] NP_525065.1, thymosin-repeated protein [*Eriocheir sinensis*] ACP19740.1, and TMSB4Y thymosin beta 4 [*Homo sapiens*] NP_004193.1.

Statistic analysis of qPCR

We analyzed the results of qPCR by One-way ANOVA, followed by a post-hoc Tukey's HSD test. The all qPCR experiments (*HsjCib* exon 1 for head, *HsjCib* exon 1 for thorax + abdomen, *HsjCib* exon 2 for head, *HsjCib* exon 2 for thorax + abdomen, *HsjCib* exon 1 for 14 d multiple tissues, and *HsjCib* exon 2 for 14 d multiple tissues) were significant by One-way ANOVA ($p < 10^{-6}$). The multiple combinations by Tukey's HSD were in Supplemental Table 1.

RNAi experiment of *HsjCib*

We tried RNA interference (RNAi) to test phenotypic consequences after *HsjCib* repression. Double-strand RNA (dsRNA) was designed for the same region with northern/Southern hybridization (Fig. 3B). Sense and antisense single-strand RNA (ssRNA) were *in vitro*-transcribed with SP6 and T7 polymerase (Clontech) from a gene fragment cloned within pGEM T-vector (Promega). DsRNA was prepared by mixing of same amounts of sense and antisense ssRNA, followed by heating (75˚C, 5min) and cooling (room temperature). Pseudergates were treated with pyriproxyfen by the ingestion method, then a week later, dsRNA was injected (1 μg or 10 μg dsRNA with PBS/individual). For negative controls, PBS and dsRNA of GFP were also injected. After molts into presoldiers, their morphologies were observed by morphometrics (45 individuals from each category, measured head width and mandible length [46], and by paraffin sections. No clear difference was found (data not shown). The actual repression of *HsjCib* expression was confirmed by qPCR (the amount of *HsjCib* transcripts was 13% of that observed in a PBS-injected negative control).

There are some possible reasons why we could not detect any RNAi effect.

We designed dsRNA in the same region as the hybridization probes (Fig. 3B), and it could have repressed both types of isoforms. If the isoforms have functions antagonistic to each other, the repression of both isoforms might conceal a phenotype. Because of the nature of the RNAi mechanism and the extreme sequence similarity among *HsjCib* isoforms, it is difficult to design dsRNA to repress one type preferentially. Also, the results could have been caused by incomplete repression of the transcript, compensation by other actin-binding proteins (such as Profilin, ADF/Cofilin, and CAP), or a phenotype too subtle to be detected by morphological observation. Because of those unknown factors and limitations of our experimental attempts, we could not conclude the morphogenetic function of *HsjCib* by RNAi experiments.
