# Supplementary material for: The homolog of Ciboulot in the termite (Hodotermopsis sjostedti): a multimeric β-thymosin involved in soldier-specific morphogenesis
Source: BMC Dev Biol. 2010 Jun 8;10:63. doi: 10.1186/1471-213X-10-63 (PMC2896938; doi:10.1186/1471-213X-10-63)
Supplement: Additional file 2 — Supplemental Fig. 1. Phylogenetic analysis of HsjCib and ciboulot homologs from other animals. [file 1471-213X-10-63-S2.PDF]

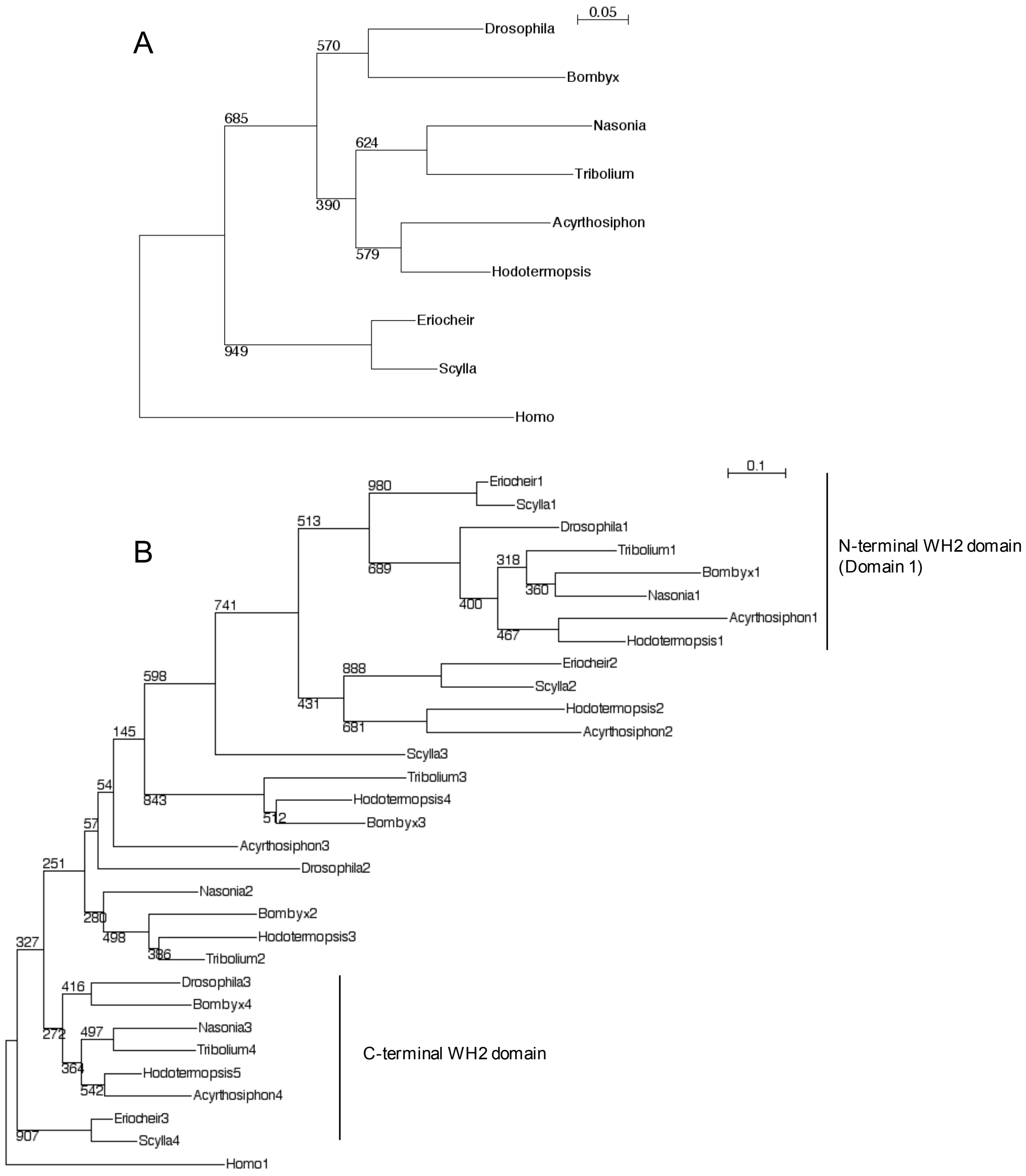

Supplemental Figure 1.

A, A neighbor-joining phylogenetic tree based on Ciboulot/Multimeric β-thymosin protein sequences. *Drosophila* (fruit fly), *Bombyx* (silkmoth), *Nasonia* (parasitoid wasp), *Tribolium* (flour beetle), *Acyrthosiphon* (pea aphid), *Hodotermopsis* (damp-wood termite), *Eriocheir* (mitten crab), *Scylla* (mud crab), *Homo* (Human). The numbers on nodes indicate bootstrap values for 1000 counts. The bar indicates genetic distance of 0.05.

B, A neighbor-joining phylogenetic tree based on WH2 domains from Ciboulot/Multimeric β-thymosin protein sequences. Numbers after genus names indicate individual WH2 domains. The most N-terminal domains are clustered together with other members respectively. The same tendency was the case in the most C-terminal domains. Intermediate domains seem to be evolved through multiple duplication/deletion events, and evolutionary relationships are not clear. The numbers on nodes indicate bootstrap values for 1000 counts. The bar indicates genetic distance of 0.10.
