# Supplementary material for: The homolog of Ciboulot in the termite (Hodotermopsis sjostedti): a multimeric β-thymosin involved in soldier-specific morphogenesis
Source: BMC Dev Biol. 2010 Jun 8;10:63. doi: 10.1186/1471-213X-10-63 (PMC2896938; doi:10.1186/1471-213X-10-63)
Supplement: Additional file 3 — Supplemental Table 1. Summary of Tukey's HSD test for qPCR results. [file 1471-213X-10-63-S3.PDF]

## Supplemental Table 1. Tukey's HSD tests for qPCR datasets.

A, Comparison among stages.

| | Head | | Abdomen+Thorax | |
|---|---|---|---|---|
| | Cib Exon1 | Cib Exon 2 | Cib Exon 1 | Cib Exon 2 |
| PE,6h | | | | |
| PE,24h | | | | |
| PE,3d | | | | |
| PE,7d | ** | | * | |
| PE,14d | ** | | ** | * |
| PE,PS | | * | | |
| PE,S | ** | ** | | |
| PE,SM | | ** | ** | ** |
| PE,N | | | | |
| PE,LN | | | ** | ** |
| PE,A | | | | ** |
| 6h,24h | | | | |
| 6h,3d | | | | |
| 6h,7d | | ** | ** | |
| 6h,14d | ** | ** | ** | * |
| 6h,PS | | | | |
| 6h,S | * | ** | | |
| 6h,SM | | ** | ** | ** |
| 6h,N | | | | |
| 6h,LN | | | ** | ** |
| 6h,A | | | | ** |
| 24h,3d | | | | |
| 24h,7d | | ** | ** | |
| 24h,14d | | ** | ** | ** |
| 24h,PS | ** | | | |
| 24h,S | | ** | | |
| 24h,SM | | ** | ** | ** |
| 24h,N | | | | |
| 24h,LN | ** | | ** | ** |
| 24h,A | * | | | ** |
| 3d,7d | | * | ** | |
| 3d,14d | * | * | ** | * |
| 3d,PS | * | | | |
| 3d,S | | ** | | |
| 3d,SM | | ** | ** | ** |
| 3d,N | | | | |
| 3d,LN | * | | ** | ** |
| 3d,A | | | | ** |
| 7d,14d | | | ** | |
| 7d,PS | ** | ** | ** | |
| 7d,S | | ** | ** | |
| 7d,SM | | | ** | ** |
| 7d,N | * | * | ** | |
| 7d,LN | ** | * | | ** |
| 7d,A | ** | | | * |
| 14d,PS | ** | ** | ** | * |
| 14d,S | | ** | ** | ** |
| 14d,SM | ** | | | |
| 14d,N | ** | * | ** | |
| 14d,LN | ** | * | ** | ** |
| 14d,A | ** | | ** | |
| PS,S | ** | ** | | |
| PS,SM | | ** | ** | ** |
| PS,N | | | | |
| PS,LN | | | ** | ** |
| PS,A | | * | | ** |
| S,SM | * | * | ** | ** |
| S,N | ** | ** | | |
| S,LN | ** | ** | ** | ** |
| S,A | ** | ** | | ** |
| SM,N | | ** | ** | ** |
| SM,LN | | ** | ** | ** |
| SM,A | | ** | ** | |
| N,LN | | | ** | ** |
| N,A | | | | * |
| LN,A | | | ** | ** |

B, Comparison among 14d tissues.

| | 14d | |
|---|---|---|
| | Exon 1 | Exon 2 |
| Mandible,Brain | ** | |
| Mandible,Muscle | ** | ** |
| Mandible,Epidermis (H) | ** | ** |
| Mandible,Leg | | |
| Mandible,Fat body | ** | ** |
| Mandible,Epidermis (A) | | |
| Mandible,Gut | ** | |
| Brain,Muscle | | ** |
| Brain,Epidermis (H) | | ** |
| Brain,Leg | ** | |
| Brain,Fat body | | ** |
| Brain,Epidermis (A) | ** | |
| Brain,Gut | | |
| Muscle,Epidermis (H) | | ** |
| Muscle,Leg | ** | ** |
| Muscle,Fat body | | ** |
| Muscle,Epidermis (A) | * | ** |
| Muscle,Gut | | |
| Epidermis (H),Leg | ** | ** |
| Epidermis (H),Fat body | | ** |
| Epidermis (H),Epidermis | ** | ** |
| Epidermis (H),Gut | | ** |
| Leg,Fat body | ** | ** |
| Leg,Epidermis (A) | | |
| Leg,Gut | * | |
| Fat body,Epidermis (A) | ** | ** |
| Fat body,Gut | | ** |
| Epidermis (A),Gut | | |

Double asterisks (**) , $p < 0.01$
Single asterisk (*) , $p < 0.05$
